# Does selection for growth rate in broilers affect their resistance and tolerance to *Eimeria maxima*?

**DOI:** 10.1016/j.vetpar.2018.06.014

**Published:** 2018-07-15

**Authors:** Panagiotis Sakkas, Idiegberanoise Oikeh, Damer P. Blake, Matthew J. Nolan, Richard A. Bailey, Anthony Oxley, Ivan Rychlik, Georg Lietz, Ilias Kyriazakis

**Affiliations:** a School of Natural and Environmental Sciences, Newcastle University, Newcastle upon Tyne, NE1 7RU, UK; b Department of Pathobiology and Population Sciences, Royal Veterinary College, University of London, North Mymms, AL9 7TA, UK; c Aviagen Ltd., Newbridge, Edinburgh, EH28 8SZ, UK; d Human Nutrition Research Centre, Institute of Cellular Medicine, Newcastle University, Newcastle upon Tyne, NE2 4HH, UK; e Veterinary Research Institute, Hudcova 70, 621 00, Brno, Czech Republic

**Keywords:** Coccidiosis, Eimeria maxima, Broiler, Genetic selection, Resistance, Tolerance, Growth rate, Bone mineralisation

## Abstract

Chickens exhibit varied responses to infection with *Eimeria* parasites. We hypothesise that broilers selected for increased growth rate will show lower resistance and tolerance to a coccidian challenge. 288 chickens of fast (F) or slow (S) growing lines were inoculated with 0 (control), 2500 (low-dose), or 7000 (high-dose) sporulated *E. maxima* oocysts at 13 days of age in two consecutive rounds. Gain and Intake were measured daily and their values relative to BW at the point of infection were calculated over the pre-patent (days 1–4 post-infection), acute (d5–8 pi), and recovery (d9–12 pi) phases of infection to assess the impact of infection. Levels of plasma carotenoids, vitamins E and A, long bone mineralisation, caecal microbiota diversity indices, and histological measurements were assessed at the acute (d6 pi) and recovery stage (d13 pi). In addition, we measured the levels of nitric oxide metabolites and the number of parasite genome copies in the jejunumat d6pi. In absolute terms F birds grew 1.42 times faster than S birds when not infected. Infection significantly reduced relative daily gain and intake (*P < *0.001), with the effects being most pronounced during the acute phase (*P <* 0.001). Levels of all metabolites were significantly decreased, apart from NO which increased (*P <* 0.001) in response to infection on d6pi, and were accompanied by changes in histomorphometric features and the presence of *E. maxima* genome copies in infected birds, which persisted to d13pi. Furthermore, infection reduced tibia and femur mineralisation, which also persisted to d13pi. Reductions in measured variables were mostly independent of dose size, as was the level of parasite replication. The impact of infection was similar for S and F-line birds for all measured parameters, and there were no significant interactions between line x dose size on any of these parameters. In conclusion, our results suggest that line differences in productive performance do not influence host responses to coccidiosis when offered nutrient adequate diets.

## Highlights

• We measured tolerance and resistance to an *Eimeria maxima* infection of 2 broiler lines differing in their growth rate.
• Infection induced typical symptoms of coccidiosis in relation to tolerance accompanied by parasite replication in the jejunum.
• Lines did not differ in their resistance or tolerance.
• Bone mineralisation was penalised by infection with effects being more pronounced at the recovery stage.

## Graphical abstract

**Figure:**
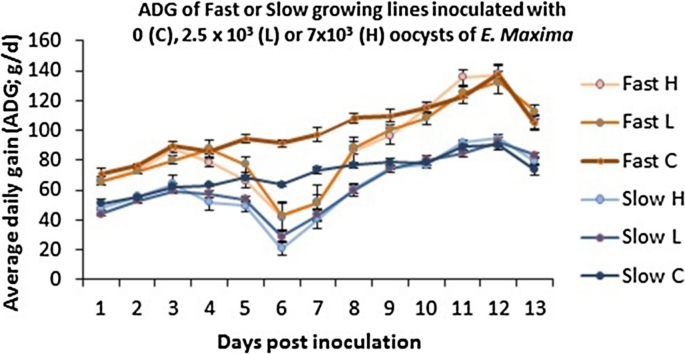

## 1 Introduction

Genetic selection for production traits, to meet increased requirements for chicken meat, has been applied to broiler chickens at an unprecedented rate (Siegel, 2014; Tixier-Boichard et al., 2012; Zuidhof et al., 2014). Such an emphasis on productive traits may have compromised the ability of modern broilers to cope with metabolic and skeletal disorders (Dawkins and Layton, 2012; Julian, 1998) and infectious pathogens (Cheema et al., 2003; Yunis et al., 2000). This raises concerns amongst the general public and have led, for example, the Dutch Organisation of Retailers to take the strategic decision that they will only sell chicken meat from slow-growing animals. Similar trends appear in other parts of the European Union (van der Aar et al., 2016).

The hypothesis is that when resources are limited, as is in the case of most health challenges, birds from lines selected for productivity will continue to direct these resources to productive rather than functional traits, such as the ability to cope with disease (Coop and Kyriazakis, 1999). This is a consequence of the genetic drive for greater productivity (Rauw, 2012). Here, we used two modern broiler lines that have been selected for different growth rates to test the hypothesis that selection for growth will penalise bird resistance to parasite infection to a greater extend (Coop and Kyriazakis, 1999). A lower level of resistance could potentially affect markers of tolerance, such as the magnitude and duration of pathogen induced anorexia, in such a way that less resistant hosts could show a delayed induction of anorexia, which is of longer duration and of smaller magnitude (Doeschl-Wilson et al., 2009; Lough et al., 2015). Host resistance has been defined as the mechanism by which the entry and/or the replication of pathogens within the host is restricted, with tolerance defined as the host’s ability to limit the detrimental effect of pathogens on performance without necessarily affecting pathogen burden (Doeschl-Wilson and Kyriazakis, 2012; Lough et al., 2015; Rauw, 2012). Although in broilers monospecific coccidian infections rarely occur in the field, a controlled coccidial infection is a good model, to test our hypothesis as the main effects are a reduction in food intake (Kipper et al., 2013; Preston-Mafham and Sykes, 1970) and absorption of nutrients (Persia et al., 2006; Preston-Mafham and Sykes, 1970; Su et al., 2014), leading to reduced availability of nutrient resources. We used infection with *Eimeria maxima* to test our hypothesis, one of the most commonly encountered coccidia spp. The magnitude of its effects depends on the degree of tissue damage and inflammation (Lillehoj and Trout, 1996; Williams, 2005), typically occuring around the period of maximum parasite schizogony and gametogony (Hein, 1968), which coincides with shortening of the villi and enlargement of crypts.

To assess tolerance we measured performance over the course of infection. In addition, we implemented two sampling points, one at the acute ((d6 post-infection (pi)) and one at the recovery stage of infection (d13pi) to measure plasma levels of lutein and zeaxanthin, which are the major carotenoids in cereal grains (Humphries and Khachik, 2003) and fat-soluble vitamins retinol (vitamin A) and α-tocopherol (vitamin E). Reduced plasma levels of both carotenoids and fat-soluble vitamins may serve as indicators of intestinal epithelial damage and may be used as markers of severity for coccidial infections (Allen et al., 2004; Allen and Fetterer, 2002a; Singh and Donovan, 1973). Furthermore, histological measurements were carried to directly assess the level of damage induced to the intestinal mucosa. Fast and slow growing broilers may differ on the level of long bone mineralisation (Williams et al., 2004) and coccidiosis has been shown to affect aspects of bone development (Fetterer et al., 2013). To that end we also assessed long bone mineralisation at both d6 and d13pi. Plasma levels of nitric oxide (NO) metabolites were also assessed at the acute stage of infection as they constitute a marker of the severity of coccidial infections (Allen, 1997a,b). They facilitate parasite killing (Lillehoj and Li, 2004), but their excessive production contributes to the pathology of *E. maxima* (Allen and Fetterer, 2002b) infections due to oxidative damage and their concentration is negatively correlated with average daily gain (ADG) and carotenoid concentration at d6pi (Zhu et al., 2000). Even though *E. maxima* does not replicate in the caeca it was hypothesised that infection in the small intestine might impact the caecal microbiota due to reduced nutrient absorption resulting in increased nutrients in the caeca, whilst differences between genetic lines of chicken have been previously observed (Schokker et al., 2015). In assessing the differences in resistance of our treatment groups, we estimated the number of parasite genome copies in the jejunum, the primary site of *E. maxima* colonisation and replication, at the peak of parasite replication (*i.e.* d6pi; (Blake et al., 2006)) and by proxy accounting for all possible underlying immune responses.

## 2 Materials and methods

### 2.1 Chicken management

All procedures were conducted under the UK Animals (Scientific Procedures) Act 1986 and EU Directive 2010/63/EU for animal experiments and carried out under Home Office authorization (P441ADF04). The experiment was conducted over two rounds, separated by 6 weeks. Each round consisted of 72 male day-old chicks of a fast-growing line (Ross 308, F), and an equal number of a slow growing line (Ross Ranger Classic, S). All birds were obtained from the same hatchery and had parents subjected to the same husbandry regime. Furthermore, the same parent stock flocks were used for each of the two lines which aged 37 and 43 weeks of age for round A and B, respectively. The growth potential of these lines differs by approximately 25%, according to the performance objectives of the breeding company. Lines F and S originate from the same paternal lines but different maternal lines; growth rate is not part of the selection criteria for the maternal lines of the S line.

Birds were housed in a windowless, thermostatically controlled room in 24 circular pens with a diameter of 1.2 m (1.13 m^2^). Pens were equipped with tube feeders and bell-drinkers, and wood shavings were used as litter to a depth of 5 cm. Birds had *ad libitum* access to feed and water throughout the trial. The temperature within the pen was monitored daily and maintained to meet recommendations for spot brooding (Aviagen, 2014b), starting at 34 °C at chick placement and was gradually reduced to 20 °C by 25 days of age. Light intensity at pen level ranged from 180 to 220 lux, while a lighting schedule of 23L:1D was applied for the first 7 days of age and switched to 18L:6D for the remainder of the trial.

Starter (d0–10) and grower (d11–26) diets were manufactured according to Aviagen nutrition specifications (Aviagen, 2014a) and were offered to both lines (Table 1). The starter diet was offered in crumb form and the grower in pelleted form.

**Table 1:** Ingredient and calculated chemical composition of the starter (d0–10) and grower (d11–26 post-hatch) diets.

Item	Starter	Grower
**Ingredient (%)**		
Wheat	47.8	51.5
Soybean meal (48 % CP)	32	25.2
Corn	10	10
Soybean full fat	4.0	7.0
Dicalcium phosphate	1.89	1.66
Soy crude oil	1.84	2.32
Limestone	0.64	0.59
Vitamin and mineral premix^1^	0.4	0.4
DL methionine	0.33	0.30
L-Lysine	0.27	0.24
Sodium bicarbonate (27 %)	0.21	0.19
Sodium chloride (39 %)	0.19	0.20
L-Threonine	0.14	0.12
Choline chloride (60 %)	0.05	0.05
L-Valine	0.03	0.02
Xylanase^3^	0.02	0.02

**Calculated nutrient composition (%)**		
ME (kcal/kg)	3,000	3,100
Crude protein	23.5	21.7
Crude fat	4.37	5.41
Calcium	0.96	0.87
Phophorus	0.76	0.70
Available phosphorus	0.48	0.44
Ash	5.23	4.78

^1,2^Provided per kilogram of feed vitamins, minerals and digestible AA according to Aviagen Nutrient specifications (Aviagen, 2014a).

^3^Ronozyme® WX, DSM Nutritional Products Ltd.

### 2.2 Experimental design and inoculations

This experiment followed a 3 × 2 factorial design with coccidian infection and bird line as the independent variables, while the experimental round was treated as a blocking factor. Upon arrival, day-old chicks of each line were randomly assigned to one of three treatment groups. Each group consisted of 8 replicate pens, and initial stocking density was 6 birds per pen. Birds were orally inoculated at 13 days of age (experimental day 0) with a single 0.5 ml dose of H_2_O (control group, C), 2500 (low-dose group, L) or 7000 (high-dose group, H) sporulated *E. maxima* oocysts of the Weybridge laboratory reference strain. Bird weight was measured at placement and bird weight and feed intake at 13 days of age (d0pi) and daily from day 1–13 pi.

### 2.3 Sampling

On d6 and d13pi, a randomly selected bird from each pen was weighed, bled from the wing vein, and then culled by lethal injection with sodium pentobarbital (Euthatal®, Merial Harlow, United Kingdom). Blood was placed in a 5 ml sodium heparin plasma tube (BD Vacutainer, SST II Advance Plus Blood Collection Tubes, Plymouth, United Kingdom). Samples were immediately placed on ice. Within 1.5 h of collection, each sample was centrifuged for 10 min at 1500× *g*/4 °C. Aliquoted plasma samples were stored at −80 °C, pending analyses.

During necropsy, 5 cm of intestinal tissue centred on Meckel’s diverticulum, the primary site of infection by *E. maxima* (Long et al., 1976), was excised and opened longitudinally, the digesta carefully removed, and the tissue submerged in 5 ml of RNAlater® (Life Technologies, Carlsbad, CA, USA). Samples were stored at −80 °C, pending further analyses. Also, 2 × 1 cm segments, one from the duodenal loop and one 2.5 cm upstream from Meckel's diverticulum, were sampled from birds dissected on d6 and d13pi and were fixed in 10% buffered formalin for morphometric analysis. Finally, the distal ends of the caeca were cut, and caecal contents were isolated in Eppendorf tubes, immediately stored at -20 °C, and transferred to −80 °C within 1 h from collection. Then, the right tibia and femur were dissected, defleshed and stored in airtight individually labelled polyethylene bags at −20 °C.

### 2.4 Sample analysis

#### 2.4.1 Quantitative real-time PCR (qPCR)

Using predicted genome sizes of 46.2 Mbp for *E. maxima* (Reid et al., 2014) and 1.2 Gbp for *G. domesticus* (Furlong, 2005), and the method of Blake et al. (2008) to extract total genomic DNA (gDNA) from sporulated *E. maxima* oocysts and uninfected chicken intestinal tissue, ten-fold DNA dilution series were created using previously described methods (Blake et al., 2006; Nolan et al., 2015). For quantifying *E. maxima* genome copy number, the primers Ema_qPCRf (forward: 5′-TCG TTG CAT TCG ACA GAT TC-3′) and Ema_qPCRr (reverse: 5′-TAG CGA CTG CTC AAG GGT TT-3′), targeting 138 base pairs of the Microneme Protein 1 (MIC1) gene, were used (Blake et al., 2006). For normalization, we used the primers actbF (forward: 5′-GAG AAA TTG TGC GTG ACA TCA-3′) and actbR (reverse: 5′- CCT GAA CCT CTC ATT GCC A -3′), which amplify 152 base pairs of the chicken cytoplasmic beta-actin (*actb*) gene, according to a previously employed protocol (Nolan et al., 2015). Total gDNA was extracted from excised intestinal tissue using a DNeasy® Blood and Tissue kit (Qiagen, Hilden, Germany), according to the manufacturer’s protocol. In brief, RNAlater® was removed from the frozen tissue, which was then weighed and immersed in an equal w/v of Qiagen tissue lysis buffer. Each sample was homogenised with a Qiagen TissueRuptor and the equivalent of ≤ 25 mg of the homogenate added to a sterile 1.5 ml microcentrifuge tube. Genomic DNA was then extracted according to manufacturer’s instructions, and stored at -20 °C until analysis.

Quantitative real-time PCR was performed with a CFX96 Touch® Real-Time PCR Detection System (Bio-Rad Laboratories, Hercules, California, USA). Amplification of each sample was performed in triplicate in a 20 μl volume containing 1 μl of total gDNA, 300 nM of each primer, 10 μl of SsoFast™ EvaGreen® Supermix (Bio-Rad Laboratories), and 8.9 μl of DNase/RNase free water (Gibco™, Life Technologies, Karlsruhe, Germany). Cycling qPCR conditions were 95 °C/2 m (enzyme activation/initial denaturation), followed by 40 cycles of 95 °C/15 s (denaturation), 60 °C/30 s (annealing/extension), followed by melt analysis of 65–95 °C at increments of 0.5 °C/0.5 s. Assays were performed in white hard-shell® 96-well PCR plates (Bio-Rad Laboratories) sealed with Thermo Scientific adhesive sealing sheets and included the respective gDNA dilution series (standards) and no template controls (NTC). Calculation of copy number of each qPCR target was performed with the software CFC Manager v.3.1 (Bio-Rad Laboratories) according to the slope and intercept of the corresponding reference dilution series. Normalization of the predicted parasite genome copy number was performed by comparison to the estimated host genome copy number. Parasite genome copy number was calculated based on the normalised parasite copy number/μl. The average, standard deviation, and relative standard deviation of quantification cycle data derived from triplicate qPCR amplification of each sample were calculated, and the efficiency (E) of each qPCR assay was determined using CFC Manager v.3.1.

#### 2.4.2 Carotenoids, vitamin A and E

Retinyl acetate and echinone were used as internal standards. Retinoid standards (> 95% *all-trans* isomers) and α-tocopherol were purchased from Sigma-Aldrich while carotenoid standards were from CaroteNature GmbH (Ostermundigen, Switzerland). HPLC-grade acetonitrile, ethanol, methanol, chloroform, hexane and triethylamine were purchased from Fisher Scientific (Loughborough, UK). Butylated hydroxytoluene (BHT) was obtained from Sigma-Aldrich. All procedures were undertaken under orange lighting to avoid analyte degradation. For the preparation of stock solutions, retinol and Vit E were dissolved in ethanol with 0.1% BHT, while lutein and zeaxanthin were dissolved in chloroform with 0.1% BHT. The concentrations of individual calibration standard solutions were confirmed by measuring the absorption in ethanol with a UV spectrophotometer. Internal standards were prepared in ethanol containing 0.01% BHT. One hundred μl of each plasma sample was diluted in 100 μl of water to which 200 μl of internal standard in ethanol was added. Two ml of hexane was added to each sample and samples were vortexed in an orbital shaker for 10 min and then centrifuged at 1500× *g* for 5 min. Following centrifugation, the upper hexane phase was transferred to clean glass tubes, and samples were re-extracted with a further 2 ml of hexane. Hexane was evaporated under a nitrogen stream, and residues were redissolved in 100 μl of ethanol and transferred to amber glass vials with inserts (Fisher Scientific). Ten μl of sample extract was injected for the analysis using a Shimadzu HPLC System *via* PDA detection, according to previously described methodology (Liu et al., 2011). The concentrations of vitamin E, lutein, zeaxanthin, and echinenone were quantified at 450 nm, while vitamin A and retinyl acetate were measured at 325 nm.

#### 2.4.3 Nitric oxide metabolites

Plasma concentrations of NO metabolites (NO_2_^−^ and NO_3_^−^), were analysed using previously described methods (Qadir et al., 2013). Spiking solution was prepared from 5 mM sodium nitrate-^15^N and 0.05 mM sodium nitrite-^15^N (Cambridge Isotope Laboratories, Inc. Andover, MA, USA) and used as an internal standard. One-hundred microliters of plasma/sample, 100 μl of spiking solution, 20 μl of 2,3,4,5,6-pentafluorobenzyl bromide (Sigma-Aldrich), and 800 μl of acetone (VWR, Lutterworth, Leicestershire, UK) were pipetted into Falcon™ round-bottom polystyrene tubes and placed in a heating block at 50 °C/120 min. Following incubation, acetone was evaporated under a nitrogen stream for 10 min. Samples were then allowed to cool before 2 ml of toluene (Fisher Scientific UK Ltd) was added to each tube and the tubes vortexed for 15 s. Subsequently, 1 ml of distilled H_2_O was pipetted into each tube, and the samples were re-vortexed twice for 15 s with a rest of 15 s in between. Using glass Pasteur pipettes, the top layer was transferred into amber glass vials and stored at -20 °C pending GCMS-analysis. Other variables such as column type and ionisation temperatures were as described by Tsikas (2000).

#### 2.4.4 Histology

Formalin-fixed intestinal sections from the duodenum and jejunum were dehydrated through a series of graded ethanol baths followed by xylene in a Shandon™ Excelsior™ ES Tissue Processor (Thermo Fisher Scientific Inc., Waltham, Massachusetts), before being embedded in paraffin wax, sectioned at 4 μm and stained with hematoxylin/eosin. Histological sections were examined under a Zeiss Primostar light microscope and images were captured using ZEN imagine software (Zeiss Germany, Oberkochen, Germany). Images were viewed to measure morphometric features of the intestinal structure at 10× magnification. From sections, the villus height and the crypt depths were determined using ImageJ (NIH) software (Schneider et al., 2012). The villus height was estimated by measuring the vertical distance from the villus tip to the villus-crypt junction for 10 villi/section, and the crypt depth by the vertical distance from the villus-crypt junction to the lower limit of the crypt, for 10 corresponding crypts/section.

#### 2.4.5 Microbiota composition

Microbiota composition was determined by sequencing of the V3/V4 variable region of 16S rRNA genes as described previously (Polansky et al., 2016; Varmuzova et al., 2016). The resulting sequences were classified by RDP Seqmatch with an OTU (operational taxonomic units) discrimination level set to 97% using Qiime software. Shannon's and Simpson’s indices for the comparison of microbiota diversity and Chao 1 index were calculated by Qiime.

#### 2.4.6 Bone mineralisation

Defleshed femur and tibia bones were thawed at 4 °C in a walk-in fridge overnight and were equilibrated to room temperature on the following day. Following that, they were subjected to a 3-point break test using an Instron testing machine (Instron 3340 Series, Single Column-Bluehill, Norwood, USA). The testing support consisted of an adjusTable 2-point block jig, spaced at 30 mm for both tibia and femur bones. The crosshead descended at 5 mm/min until a break was determined by measuring a reduction in force of at least 5%. Following breaking strength determination bones were split in two, and the bone marrow was manually removed. Subsequently, bones were soaked in petroleum ether for 48 h for lipid removal and then placed in an oven at 105 °C for 24 h. The dry bone weight was recorded, and samples were ashed for 24 h at 600 °C for the determination of ash weight (g) and ash content (%).

**Table 2:** Effects of line, dose and their interaction on performance parameters in broiler chickens of either fast or slow growing lines, inoculated with 0 (Control), 2.5 × 10^3^ (Low) or 7 × 10^3^ (High) sporulated *E. maxima* oocysts over the period post infection (d0-d13pi) (LS means with SEM).

		BW d0 (g)	BW d13 (g)	ADG (g/d)	ADFI (g/d)	FCR
Line						
	Slow	371	1220	65.3	93.4	1.43
	Fast	479	1676	92.1	122.7	1.34
	SEM	3.4	14.4	1.01	1.17	0.009
Dose						
	Control	427	1541^a^	85.7^a^	114.2^a^	1.34^a^
	Low	422	1401^b^	75.3^b^	104.2^b^	1.39^b^
	High	427	1402^b^	75.0^b^	105.7^b^	1.42^b^
	SEM	4.2	17.6	1.24	1.44	0.012
Line × Dose						
Slow	Control	373	1296	71.0	98.6	1.39
	Low	369	1182	62.6	90.2	1.44
	High	373	1181	62.1	91.5	1.47
Fast	Control	481	1786	100.4	129.8	1.29
	Low	475	1619	88.1	118.3	1.34
	High	481	1624	87.9	120.0	1.37
	SEM	5.9	24.9	1.8	2.03	0.016
Source		*Probabilities*				
Line		*<0.001*	*<0.001*	*<0.001*	*<0.001*	*<0.001*
Dose		0.599	*<0.001*	*<0.001*	*<0.001*	*<0.001*
Line × Dose		0.972	0.522	0.476	0.716	0.981

^a-b^Means within a column that do not share a common superscript are significantly different (*P* < 0.05).

Abbreviations: BW, body weight; AD, Gaverage daily gain; AD, FIaverage daily feed intake; FCR, feed conversion ratio.

### 2.5 Calculations and statistics

**Fig. 1:**
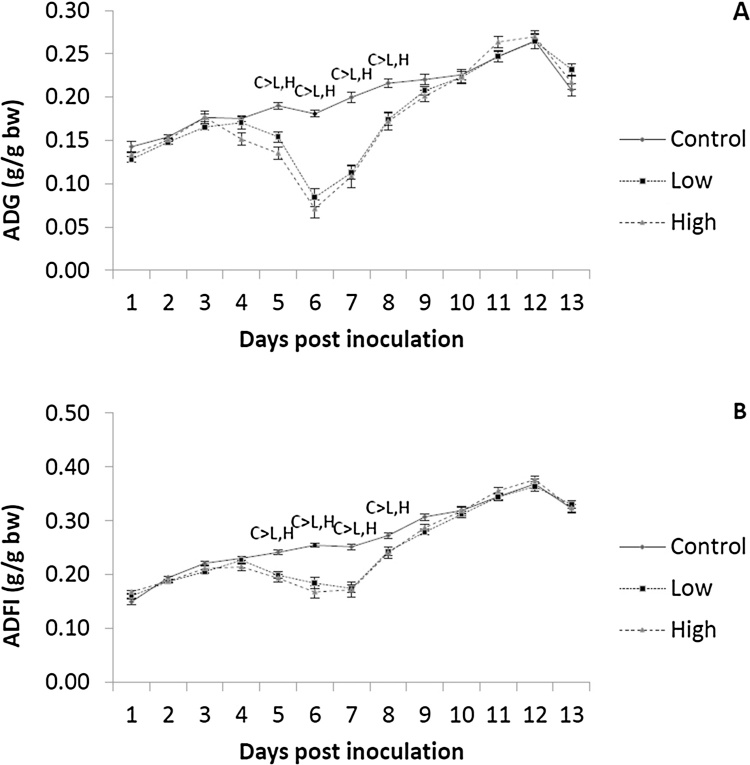
Daily ADG (A) and ADFI (B) as a proportion of BW (g/g) at d0 post inoculation (pi) with 0 (Control), 2,5 × 10^3^ (L) or 7 × 10^3^ (H) sporulated *E. maxima* oocysts over d1-13pi. Superscript values indicate significant differences between means of the control (C), low (L), and (H) doses (*P* < 0.05).

All statistical analyses were conducted in SAS 9.4 (SAS Institute, Cary, NC). For all statistical assessments pen was considered the experimental unit. Average daily feed intake (ADFI), average daily gain (ADG) (g/d) and feed conversion ratio (FCR, ADFI/ADG) were calculated over the period post-infection and were analysed with dose, line and trial as fixed factors and the interaction between line and dose with the general linear model procedure (PROC GLM) (Table 2). To account for the *a priori* differences in performance between the broiler lines, ADFI, and ADG data were expressed a proportion of BW on d0pi (ADG/BW and ADFI/BW in g/d/g). These were analysed with the repeated measurements mixed procedure (PROC MIXED). The model included dose, line, day and round as fixed factors, the 2-way interactions between dose and line, dose and day and the three-way interaction between dose, day and line. Covariance structures were chosen based on the lowest value for the Akaike and Bayesian information criteria. Based on dpi that a reduction of ADFI was observed (Fig. 1) (see below), effects of infection on ADFI/BW, ADG/BW and FCR were calculated over the pre-patent (d1–4), acute (d5–8), and recovery (d9–12 pi) periods of infection with PROC GLM using the same model as performance data over the whole period pi (Table 3). Single time point data included plasma concentrations of zeaxanthin, lutein, vitamins E and A, histological and bone measurements, Shannon, Simpson and Chao 1 indices deriving from one bird per pen dissected on d6 or d13pi, as well as *E. maxima* genome copy numbers and nitric oxide metabolites (NO) obtained at d6pi. Histological and bone measurements, apart from ash percentage and villi length/crypt depth ratio (VCR), were expressed as a proportion of BW of dissected birds. This was done to account for the size difference between F and S growing birds and between control and infected birds. Expressing bone variables as a proportion of BW has been previously used in studies comparing genotypes differing in their growth potential (Shim et al., 2012). These were analysed with PROC GLM with dose, line and round as fixed factors and the interaction between dose and line. For *E. maxima* genome copy numbers control birds were excluded from the model as their value was effectively 0. For all statistical procedures, the normality of the residuals was assessed with the Shapiro-Wilk test. Predicted *E. maxima* genome copy numbers were log transformed and the Simpson index values were arcsine transformed before analysis to obtain a normal distribution of the residuals. When significant differences were detected, treatment means were separated and compared by the Tukey’s multiple comparison test. Significance was determined at *P* < 0.05. All data are expressed as model-predicted least square means with the SEM.

**Table 3:** Effects of line, dose and their interaction on ADG/BW (g/d/g) and ADFI/BW(g/d/g) and FCR, during the pre-patent (d1–4), acute (d5–8), or recovery (d8–12) period of infection in broiler chickens of either fast or slow growing lines, inoculated with 0 (Control), 2.5 × 10^3^ (Low) or 7 × 10^3^ (High) sporulated *E. maxima* oocysts (LS means with SEM).

		Pre-patent			Acute			Recovery		
		ADFI/BW	ADG/BW	FCR	ADFI/BW	ADG/BW	FCR	ADFI/BW	ADG/BW	FCR
Line										
	Slow	0.194	0.149	1.30	0.217	0.144	1.57	0.326	0.226	1.44
	Fast	0.199	0.163	1.22	0.217	0.160	1.39	0.338	0.251	1.34
	SEM	0.0015	0.0018	0.011	0.0033	0.0035	0.029	0.0039	0.0030	0.009
Dose										
	Control	0.199	0.162^a^	1.23^a^	0.255^a^	0.197^a^	1.30^a^	0.335	0.239	1.40
	Low	0.195	0.153^b^	1.28^b^	0.200^b^	0.131^b^	1.54^b^	0.325	0.235	1.38
	High	0.195	0.153^b^	1.28^b^	0.196^b^	0.127^b^	1.60^b^	0.336	0.241	1.40
	SEM	0.0019	0.0022	0.014	0.0040	0.0042	0.036	0.0048	0.0037	0.012
Line × Dose										
Slow	Control	0.197	0.156	1.26	0.253	0.190	1.33	0.331	0.226	1.47
	Low	0.195	0.145	1.32	0.206	0.126	1.65	0.318	0.225	1.41
	High	0.191	0.147	1.32	0.193	0.115	1.72	0.329	0.227	1.45
Fast	Control	0.201	0.168	1.19	0.257	0.204	1.27	0.339	0.253	1.34
	Low	0.199	0.161	1.24	0.199	0.137	1.44	0.332	0.255	1.35
	High	0.195	0.159	1.23	0.195	0.139	1.48	0.342	0.246	1.34
	SEM	0.0025	0.0031	0.019	0.0057	0.0060	0.051	0.0068	0.0053	0.017
Source		*Probabilities*								
Line		*0.045*	*<0.001*	*<0.001*	0.972	*0.003*	*<0.001*	*0.045*	*<0.001*	*<0.001*
Dose		0.269	*0.008*	*0.020*	*<0.001*	*<0.001*	*<0.001*	0.210	0.546	0.479
Line × Dose		0.314	0.707	0.801	0.295	0.522	0.222	0.892	0.769	0.154

^a-b^Means within a column that do not share a common superscript are significantly different (*P* < 0.05).

Abbreviations: BW, body weight; ADG, average daily gain (g/d/g of BW at d0pi); ADFI, average daily feed intake (g/d/g of BW at d0pi); FCR, feed conversion ratio.

## 3 Results

### 3.1 Performance variables over the infection period

Main effects of line, dose and their interaction on ADG, ADFI, and FCR are presented in Table 2. Line and parasite dose did not significantly interact for any of the performance parameters (*P >* 0.1). Parasite dose significantly affected BW (*P <* 0.001) at the end of the trial period (d13 pi) and ADG and ADFI over the period pi (*P <* 0.001), with H and L dosed birds showing smaller values in comparison to C birds and similar to each other, whilst the opposite was the case for FCR. Line significantly affected (*P <* 0.001) all performance parameters over the period post infection with F line birds being heavier than S line birds at inoculation (d0pi) and the end of the trial (d13pi).

### 3.2 Repeated measurements on daily ADFI/BW and ADG/BW

There were no significant interactions between line and dose on ADG/BW or ADFI/BW (g/d/g) (*P* > 0.1). Even when expressing values as a proportion of BW at infection F line birds continued having greater ADG (*P <* 0.001) and ADFI (*P =* 0.003) than S line birds (0.194 *vs* 0.176; SEM = 0.001 and 0.257 *vs* 0.252; SEM = 0.001, respectively). Dose affected ADG/BW and ADFI/BW (*P <* 0.001); in comparison to uninfected birds, L and H dosed birds showed significantly smaller ADG/BW (0.200 *vs* 0.177 *vs* 0.177; SEM = 0.02) and ADFI/BW (0.268 *vs* 0.247 *vs* 0.249; SEM = 0.02 for C, L, and H birds, respectively). ADG/BW and ADFI/BW were affected by the interaction between dose and day (P < 0.001); H and L dosed birds showed significantly smaller ADG/BW and ADFI/BW between d4 and d8 pi compared to the controls. Similar effects were observed for ADFI (*P* < 0.001) (Fig. 1). For this reason, the experimental period was divided into three equal periods that roughly equated to the pre-patent (d1–4pi), acute (d5–8pi), and recovery (d9–12pi) periods of infection.

### 3.3 ADG/BW, ADFI/BW and FCR during the pre-patent, acute, and recovery periods

The main effects of line, dose and their interaction on ADG/BW, ADFI/BW, and FCR over the pre-patent, acute and recovery periods post infection are presented in Table 3. Line and parasite dose did not significantly interact for any of the performance parameters on either period post infection (*P >* 0.1). Dose significantly affected ADG/BW and FCR during the prepatent and acute periods as well as ADFI/BW during the acute period (*P <* 0.05); in all cases C birds had significantly greater ADFI/BW and ADG/BW and smaller FCR than L and H dosed birds. F line birds had significantly greater ADFI/BW, ADG/BW and smaller FCR than S line birds during all periods apart from the acute period when they had similar ADFI/BW with S line birds.

### 3.4 Carotenoids, vitamin A, E, and nitric oxide metabolites

The main effects of dose, line and their interaction on the concentration of carotenoids, vitamin A, E (μm/l) and NO (μM) are presented in Table 4. Dose significantly affected the concentration (*P <* 0.001) of lutein, zeaxanthin, and vitamins A and E at d6pi, being significantly greater in control birds compared to L and H dose birds. Similar effects were observed at d13pi for lutein (*P <* 0.001), zeaxanthin (*P* = 0.007), and vitamin E (*P* = 0.030). In contrast, dose induced an opposite effect on NO at d6pi (*P* < 0.001). Bird line did not affect the concentration of any of the measured metabolites at d6 and d13pi (*P >* 0.1), apart from vitamin A (*P <* 0.001), which was greater on d13pi for S than for F line birds. Line and dose interacted for plasma vitamin E (*P =* 0.050); at d13pi control birds of the line S had significantly greater values than those of the L and H dose birds of line S and H dose birds of line F.

**Table 4:** Effects of line, dose and their interaction on plasma metabollite concentration (μm/l) in broiler chickens of either fast or slow growing lines, inoculated with 0 (Control), 2,5 × 10^3^ (Low) or 7 × 10^3^ (High) sporulated *E. maxima* oocysts (LS means with SEM).

		d6 post-infection					d13 post-infection			
		lutein	zeaxanthin	vitamin E	vitamin A	NO (μM)	lutein	zeaxanthin	vitamin E	vitamin A
Line										
	Slow	1.12	0.269	42.8	3.16	48.6	1.93	0.412	69.9	4.63
	Fast	1.19	0.285	41.9	2.95	42.9	1.86	0.402	69.2	3.84
	SEM	0.052	0.0117	1.91	0.117	3.10	0.064	0.0149	2.56	0.110
Dose										
	Control	2.27^a^	0.515^a^	93.0^a^	4.19^a^	28.7^a^	2.16^a^	0.456^a^	76.1^a^	3.97
	Low	0.65^b^	0.169^b^	17.8^b^	2.65^b^	54.2^b^	1.77^b^	0.387^b^	68.4^b^	4.34
	High	0.55^b^	0.146^b^	16.3^b^	2.32^b^	54.4^b^	1.75^b^	0.377^b^	64.1^b^	4.39
	SEM	0.064	0.014	2.34	0.143	3.79	0.079	0.0182	3.14	0.134
Line × Dose										
Slow	Control	2.25	0.505	97.0	4.23	31.9	2.27	0.476	82.3^a^	4.46
	Low	0.64	0.173	16.9	2.78	62.1	1.70	0.372	63.3^b^	4.64
	High	0.47	0.130	14.6	2.47	51.8	1.82	0.389	64.1^b^	4.79
Fast	Control	2.30	0.526	88.9	4.15	25.5	2.05	0.436	70.0^ab^	3.49
	Low	0.66	0.166	18.7	2.52	46.2	1.85	0.402	73.5^ab^	4.03
	High	0.62	0.163	18.0	2.18	57.0	1.68	0.366	64.1^b^	4.00
	SEM	0.090	0.0203	3.31	0.203	5.36	0.113	0.0258	4.44	0.190
Source		*Probabilities*								
Line		0.326	0.342	0.735	0.224	0.204	0.417	0.621	0.838	*<0.001*
Dose		*<0.001*	*<0.001*	*<0.001*	*<0.001*	*<0.001*	*<0.001*	*0.007*	*0.031*	0.066
Line × Dose		0.758	0.601	0.184	0.865	0.164	0.225	0.370	*0.050*	0.622

^a-b^Means within a column that do not share a common superscript are significantly different (*P* < 0.05).

Abbreviations: NO, Nitric oxide metabolites.

### 3.5 qPCR and histology

The main effects of dose, line and their interaction on histological measurements villi length and crypt depth (um/kg of BW at dissection), VCR and number of *E. maxima* genome copies for measurements obtained on d6 and 13pi are presented in Table 5, Table 6, respectively. Parasite genomes were not detected in samples collected from control birds at d6pi. Parasite replication on d6pi was not affected by any of the independent variables or the interaction. Dose significantly affected (*P* < 0.05) all digestive tract morphological parameters at d6pi; infected H and L birds had shorter villi and enlarged crypts and a smaller VCR in comparison to C birds, whilst being similar to each other. At d13pi, duodenal and jejunal villi length were significantly affected by dose (*P* = 0.016 and *P* = 0.008; respectively) being significantly longer (*P <* 0.05) in H and L dose birds in comparison to C birds. Crypt depth of intestinal sections, were significantly affected by dose (*P* < 0.001) being greater for H and L dose birds compared to C birds. On the other hand, VCR was significantly affected by dose (*P* < 0.05), being significantly smaller for H dose birds in the duodenum and both and H and L birds in the jejunum than C birds. Line F birds had significantly shorter villi and smaller crypt depth (*P <* 0.01) at all intestinal sites at both dpi. Line and dose interacted for crypt depth in the jejunum (*P* = 0.024) at d6pi with S birds receiving the H and L doses displaying significantly (*P <* 0.05) greater crypt depth than F line L dosed birds.

**Table 5:** Effects of line, dose and their interaction on intestinal morphology, log transformed *E. Maxima* genomy copy number (GC), Shannon, arcsine transformed Simpson and Chao 1 indices at d6 post inoculation in broiler chickens of either fast or slow growing lines, inoculated with 0 (Control), 2,5 × 10^3^ (Low) or 7 × 10^3^ (High) sporulated *E. maxima* oocysts (LS means with SEM).

		Duodenum			Jejunum				Caeca		
		VL/BW (μm/kg)	CD/BW (μm/kg)	VCR	VL/BW (μm/kg)	CD/BW (μm/kg)	VCR	*E. maxima* GC	Shannon index	Simpson index	Chao 1 index
Line											
	Slow	1699	396	5.45	761	293	3.71	5.87	6.72	1.26	9609
	Fast	1428	289	6.00	574	231	3.63	5.89	6.99	1.27	8502
	SEM	46.9	16.8	0.260	35.8	14.7	0.282	0.12	0.179	0.024	435
Dose											
	Control	1861^a^	212^a^	9.49	757^a^	125^a^	6.45^a^	*NA*	6.85	1.29	8847
	Low	1467^b^	380^b^	4.26	646^b^	311^b^	2.48^b^	5.89	6.83	1.24	9221
	High	1362^b^	436^b^	3.42	599^b^	349^b^	2.07^b^	5.87	6.87	1.27	9100
	SEM	57.4	20.6	0.32	43.8	18.0	0.34	0.12	0.219	0.029	532
Line × Dose											
Slow	Control	1957	227	9.28	836	136^c^	6.63	*NA*	7.08	1.29	7885
	Low	1669	444	3.98	732	385^a^	2.19	5.91	7.21	1.22	8675
	High	1471	517	3.10	714	359^a^	2.29	5.87	6.68	1.31	8946
Fast	Control	1765	196	9.71	677	114^c^	6.27	*NA*	6.62	1.29	9809
	Low	1265	317	4.54	561	238^b^	2.78	5.88	6.5	1.27	9767
	High	1254	354	3.74	483	340^ab^	1.85	5.87	7.08	1.23	9254
	SEM	81.2	29.1	0.451	61.9	25.5	0.487	0.17	0.310	0.041	752
Source		*Probabilities*									
Line		*<0.001*	*<0.001*	0.146	*0.001*	*0.005*	0.858	0.940	0.302	0.777	0.080
Dose		*<0.001*	*<0.001*	*<0.001*	*0.049*	*<0.001*	*<0.001*	0.880	0.988	0.491	0.876
Line × Dose		0.370	0.073	0.973	0.825	*0.024*	0.498	0.927	0.177	0.298	0.579

^a-c^Means within a column that do not share a common superscript are significantly different (*P <* 0.05).

Abbreviations: VL, villus length; CD, crypt depth; VCR, villi length: crypt depth ratio; BW, body weight at dissection.

**Table 6:** Effects of line, dose and their interaction on intestinal morphology, Shannon, arcsine transformed Simpson and Chao 1 indices at d13 post inoculation in broiler chickens of either fast or slow growing lines, inoculated with 0 (Control), 2,5 × 10^3^ (Low) or 7 × 10^3^ (High) sporulated *E. maxima* oocysts (LS means with SEM).

		Duodenum			Jejunum			Caeca		
		VL/BW (μm/kg)	CD/BW (μm/kg)	VCR	VL/BW (μm/kg)	CD/BW (μm/kg)	VCR	Shannon index	Simpson index	Chao 1 index
Line										
	Slow	1241	128	10.15	578	97	6.33	6.79	1.26	9822
	Fast	956	104	9.77	467	80	6.28	7.08	1.29	9886
	SEM	24.7	2.9	0.324	22.1	3.0	0.239	0.142	0.018	595
Dose										
	Control	1026^a^	98^a^	10.82^a^	448^a^	68^a^	7.05^a^	6.92	1.28	10245
	Low	1118^b^	120^b^	9.76^a,b^	552^b^	97^b^	6.02^b^	7.10	1.28	9557
	High	1151^b^	130^b^	9.30^b^	566^b^	100^b^	5.84^b^	6.80	1.26	9761
	SEM	30.2	3.6	0.397	27.0	3.7	0.293	0.173	0.022	729
Line × Dose										
Slow	Control	1164	110	11.02	498	74	6.92	7.36	1.24	9126
	Low	1280	137	9.63	610	107	6.20	6.93	1.31	10482
	High	1279	136	9.80	625	109	5.87	6.94	1.24	9858
Fast	Control	888	86	10.63	399	62	7.19	6.48	1.32	11364
	Low	956	102	9.88	494	88	5.84	6.66	1.25	8631
	High	1023	124	8.79	508	90	5.82	7.24	1.29	9664
	SEM	42.8	5.1	0.561	38.2	5.2	0.413	0.245	0.031	1031
Source		*Probabilities*								
Line		*<0.001*	*<0.001*	0.406	*0.001*	*<0.001*	0.887	0.164	0.282	0.940
Dose		*0.016*	*<0.001*	0.029	*0.008*	*<0.001*	*0.013*	0.489	0.809	0.792
Line × Dose		0.726	0.083	0.541	0.964	0.744	0.756	0.068	0.087	0.150

^a-c^Means within a column that do not share a common superscript are significantly different (*P <* 0.05).

Abbreviations: VL, villus length; CD, crypt depth; VCR, villi length: crypt depth ratio; BW, body weight at dissection.

### 3.6 Microbiota composition

Main effects of line, dose and their interaction on Shannon, Simpson and Chao 1 indexes are presented in Table 5, Table 6 for birds sampled on d6 and 13pi, respectively. None of the indexes was affected by any of the independent variables or their interaction.

### 3.7 Bone mineralisation

The main effects of line, dose and their interaction on femur and tibia measurements at d6 and d13 pi are presented in Table 7, Table 8, respectively. There was no significant interaction between line and dose on tibia and femur parameters both on d6pi and d13pi. The impact of infection on d6pi was evident on femur: dose significantly affected BBS/BW (*P =* 0.019) and ash percentage (*P =* 0.038), which were significantly greater for C than H and L birds. On the contrary on d13pi, dose significantly reduced all markers of tibia mineralisation; BBS, ash and ash percentage were significantly smaller for H in comparison to C birds (*P <* 0.05) whereas values of L birds were intermediate and not significantly different to either group. In addition, it affected femur ash (*P <* 0.05), which was significantly smaller for H in comparison to C birds (*P <* 0.05). Moreover, there was an effect (*P =* 0.005) on femur ash percentage being significantly smaller for H and L birds in comparison to C birds. Tibia ash weight was significantly greater for the S in comparison to the F line on both d6 (*P <* 0.01) and 13pi (*P <* 0.05), but femur ash weight only on d13pi (*P <* 0.001). On the other hand, BBS was only significantly larger (*P <* 0.05) for the femurs of the S growing line at d13pi, while they did not differ in ash percentage.

**Table 7:** Effects of line, dose and their interaction on long bone mineralisation parameters at d6 post inoculation, in broiler chickens of either fast or slow growing lines, inoculated with 0 (Control), 2,5 × 10^3^ (Low) or 7 × 10^3^ (High) sporulated *E. maxima* oocysts (LS means with SEM).

		Tibia			Femur		
		BBS/BW (N/kg)	Ash/BW (g/kg)	Ash (%)	BBS/BW (N/kg)	Ash/BW (g/kg)	Ash (%)
Line							
	Slow	212	1.13	42.6	194	0.805	44.3
	Fast	197	1.07	42.2	181	0.778	44.4
	SEM	10.0	0.018	0.45	6.2	0.0151	0.42
Dose							
	Control	213	1.09	42.8	204^a^	0.809	45.4^a^
	Low	196	1.11	42.5	172^b^	0.786	43.6^b^
	High	204	1.09	41.8	186^ab^	0.779	43.9^ab^
	SEM	12.2	0.022	0.55	7.6	0.0185	0.51
Line × Dose							
Slow	Control	209	1.12	43.3	209	0.813	45.4
	Low	205	1.15	43.0	180	0.812	43.9
	High	222	1.10	41.4	191	0.788	43.6
Fast	Control	218	1.07	42.3	198	0.805	45.5
	Low	188	1.07	42.1	163	0.759	43.4
	High	185	1.07	42.1	181	0.769	44.2
	SEM	17.3	0.032	0.77	10.8	0.0262	0.72
Source		*Probabilities*					
Line		0.296	*0.030*	0.540	0.147	0.216	0.889
Dose		0.629	0.711	0.391	*0.019*	0.477	*0.038*
Line × Dose		0.422	0.761	0.446	0.937	0.672	0.727

^a-c^Means within a column that do not share a common superscript are significantly different (*P* < 0.05).

Abbreviations: BBS, bone breaking strength; BW, body weight at dissection.

**Table 8:** Effects of line, dose and their interaction on long bone mineralisation parameters at d13 post inoculation, in broiler chickens of either fast or slow growing lines, inoculated with 0 (Control), 2,5 × 10^3^ (Low) or 7 × 10^3^ (High) sporulated *E. maxima* oocysts (LS means with SEM).

		Tibia			Femur		
		BBS/BW (N/kg)	Ash/BW (g/kg)	Ash (%)	BBS/BW (N/kg)	Ash/BW (g/kg)	Ash (%)
Line							
	Slow	191	1.15	44.9	175	0.821	43.8
	Fast	194	1.08	45.1	152	0.750	43.7
	SEM	7.2	0.019	0.21	6.4	0.0118	0.42
Dose							
	Control	215^a^	1.15^a^	45.8^a^	168	0.809^a^	45.1^a^
	Low	189^ab^	1.13^ab^	44.7^b^	162	0.794^ab^	42.7^b^
	High	174^b^	1.05^b^	44.4^b^	160	0.754^b^	43.4^b^
	SEM	8.8	0.024	0.26	7.8	0.0145	0.51
Line × Dose							
Slow	Control	210	1.19	45.4	180	0.858	45.5
	Low	200	1.19	45.2	186	0.846	42.9
	High	164	1.05	44.1	160	0.760	42.9
Fast	Control	221	1.18	46.2	157	0.760	44.8
	Low	179	1.07	44.3	139	0.743	42.4
	High	183	1.05	44.7	161	0.749	43.9
	SEM	12.4	0.032	0.37	10.9	0.0205	0.72
Source		*Probabilities*					
Line		0.764	*0.021*	0.593	*0.014*	*<0.001*	0.924
Dose		*0.007*	*0.011*	*0.001*	0.748	*0.030*	*0.005*
Line × Dose		0.237	0.182	0.052	0.104	0.052	0.424

^a-c^Means within a column that do not share a common superscript are significantly different (P < 0.05).

Abbreviations: BBS, bone breaking strength; BW, body weight at dissection.

## 4 Discussion

We tested the hypothesis that resistance and tolerance of modern broiler lines to *E. maxima* infection would be sensitive to genetic selection for growth rate. This hypothesis was based on the assumption that as resource intake during *Eimeria* infection is limited, mainly due to pathogen-induced anorexia (Kyriazakis, 2014) and reduced nutrient absorption (Preston-Mafham and Sykes, 1970), fast-growing birds would divert more resources to growth rather than to pathogen-coping functions. Furthermore, the supposition was that at increasing pathogen doses, the concomitant resource limitation would be greater for the fast-growing line. We appreciate that there are potential confounding issues when making comparisons between hosts of different size as has been pointed out previously (Coltherd et al., 2011), which may arise, for example, from the relative nutrition of the host or parasite dose given to large and small size birds. Accounting for all these factors will make for a very complex experimental design and for this reason we have opted for an experiment where the treatments imposed on both bird genotypes were similar. Contrary to our hypothesis, lines did not differ in their resistance or tolerance to *E. maxima*. In the subsequent segments of the discussion we dissect the effects of dose and line on response variables and we discuss the absence of interactive effects.

To assess variation in parasite replication as a consequence of differential resistance between fast and slow growing commercial lines, we used quantitative real-time PCR to measure parasite genome copy number in tissues surrounding Meckel’s diverticulum, which supports higher throughput analysis than conventional measurement of oocysts per gram. The approach also minimised the impact of variation related to the temporal manner of oocyst excretion (Blake et al., 2006). Quantitative real-time PCR has previously been used to define variation in parasite replication in chicken lines with a known polymorphism in their resistance to *E. maxima*, revealing the biggest differences five and six days after infection. In this study, predicted parasite copy number at d6pi in line F and S birds was similar, rejecting our hypothesis that a fast-growing line may show reduced resistance. Moreover, there were no significant differences observed among birds from line S and F given L or H doses, likely illustrating the crowding effect on parasite replication (*i.e.* parasite fecundity is reduced once a ‘threshold’ has been reached) (Williams, 2001).

Infection with *E. maxima* resulted in reductions in relative ADFI and ADG between d4–8pi. No differences in performance were observed between control and infected birds during the recovery period of infection. Thus, our results do not suggest that infected birds had the capacity for compensatory growth within the period of study. This is in accordance with results obtained in broilers infected with coccidia sp. (Gabriel et al., 2006; Preston-Mafham and Sykes, 1970; Takhar and Farrell, 1979), but on the other hand may be a reflection of the post-recovery environment and especially food composition which may define if a host is able to compensate or not (Kyriazakis and Houdijk, 2007). The observed reductions in performance during the acute stage of infection were coupled with damage to the gastrointestinal mucosa of the duodenum and jejunum at d6pi. At d13pi birds showed compensatory villi development, but the pathological effects of infection persisted as birds also displayed reduced VCR at both intestinal sites. A marked reduction in plasma carotenoid concentration levels was observed during the acute phase of infection, which is characteristic of coccidian infections affecting the proximal intestine (Allen, 1992; Hernández-Velasco et al., 2014; Zhu et al., 2000). Similar reductions were observed for vitamin E, in accordance with previous studies (Allen and Fetterer, 2002a, b). These reductions are attributed to malabsorption caused by the damage to the gastrointestinal mucosa (Allen and Fetterer, 2002a, b), leading to defective fat absorption (Adams et al., 1996; Sharma and Fernando, 1975), and to oxidation by reactive oxygen species (Allen, 1997b). In the present study, these effects persisted to d13pi for carotenoids compared to the effects on vitamin A, which may have increased in concentration as a result of its release by the liver (Harrison, 2005). We also assessed levels of NO metabolites at d6pi that were significantly elevated as a result of infection according to our expectation. Our results confirm the lack of alpha diversity reported previously where birds were inoculated with *E. tenella* (Macdonald et al., 2017; Zhou et al., 2017) or mixed infection (*E. acervulina, E. maxima, E. tenella*) (Martynova-Van Kley et al., 2012).

As far as bone mineralisation is concerned, older studies using *E. acervulina* have shown impaired calcium and mineral absorption and retention although a higher absorption efficiency has been observed during the recovery period, depending on infection severity (Takhar and Farrell, 1979; Turk, 1973). Investigations with *E. acervulina* in starter chicks (infection at d2-d8) have shown reduced bone ash, bone Ca content, or Ca:P ratio (Giraldo et al., 1987; Ward et al., 1990; Watkins et al., 1989; Watson et al., 2005; Willis and Baker, 1981). A recent investigation employing a natural co-infection with *E*. *acervulina* and *E*. *tenella,* by placing day old chicks in seeded litter has revealed that BBS is adversely affected by 21 days of age (Shaw et al., 2011). However, in that study BBS data were not adjusted for the birds’ reduced BW following infection, while the timing of effects in relation to the parasite cycle is unknown when using a natural model of infection. Finally, single species infections with either *E. acervulina* or *E. maxima* have been shown to reduce bone mineral content at d6pi, but their effects have not been investigated at later time points (Fetterer et al., 2013). Ours is the first study to investigate bone mineralisation at the acute and recovery timepoints over the course of a primary infection with an *Eimeria* sp. in broiler chicks. Effects on bone mineralisation were present across timepoints, but were more pronounced at the recovery stage. Mineralisation of the femur was affected earlier than the tibia post infection; this could be attributed to the faster mineralisation rate of the femur compared to the tibia during the initial stages of broiler growth (Applegate and Lilburn, 2002). By d13pi (d26 post-hatch), although infected birds matched the growth rates of their non-infected counterparts, their bones were less mineralised with effects being present for both long bones. This indicates, that upon recovering from a limitation of nutrient resources as a result of coccidiosis, bone development lags behind tissue accretion.

We hypothesised that a greater level of parasite infection would have a greater impact on line F birds. The observed differences between L and H dose birds were not statistically significantly different. This may be related to the relatively high pathogenicity of *E. maxima* and the magnitude of the difference between dose sizes. Comparison between sizes of infective doses administered in other studies are not of direct relevance as the age at which birds were infected, the broiler line, and *E. maxima* strain used are directly related to the pathogenicity of the dose administered (Allen, 1997a,b; Allen et al., 2005; Conway et al., 1993; Idris et al., 1997). Our results are consistent with the suggestion that over a range of infectious doses the extent of pathogen-induced anorexia is similar (Sandberg et al., 2007).

Previous studies comparing broiler genotypes in their resistance to coccidian infections have utilised genetically distant inbred populations (Allen and Lillehoj, 1998; Bumstead et al., 1995; Lillehoj and Ruff, 1987), outbred lines (Pinard-Van Der Laan et al., 1998), or lines selected for different pro-inflammatory response (Swaggerty et al., 2015) to elucidate effector mechanisms contributing to resistance, and their relationship to the magnitude of penalties observed on performance. To our knowledge, the only study which specifically compared lines differing in their performance objectives was that of Swinkels et al. (2007), where genotypes originating from distinctively different lineages of chicken were contrasted (Ross 308 *vs* Hubbard JA 957). Therefore, the present study is the first to address specifically selection for growth rate on resistance and tolerance to coccidian infections. Over the post-infection period, the two lines differed by 30% in their final body weight at d26 of age while they differed by 7% in their FCR, according to expectations.

In a recent review (Tallentire et al., 2016), summarising effects of selection for performance on the digestive physiology of broilers, it was stated that selection for growth rate has reduced the size of the gastrointestinal tract (GIT), but is accompanied by increased surface area due to greater intestinal villi size. In the present study the relative size of GIT was not assessed. However, we found that F line birds had shorter villi than S line birds when villi length was expressed relative to BW. Coupled with the smaller FCR of F line birds, this finding suggests that these birds have the ability to absorb nutrients more efficiently. In the presence of infection, one would expect a smaller impact on FCR in F than S line birds attributed to the proportionally smaller GIT and the concomitant smaller energetic and nutrient costs which would accompany the repair of damaged intestinal tissue (Sandberg et al., 2007). Although, a statistically significant interaction was not attained for FCR, the percentage increase over the acute period of infection was greater for S than F line birds (≈ 26 *vs* 15%) in relation to their non-infected counterparts. A larger number of replicates may have supported the aforementioned notion. Irrespective of differences in intestinal architecture, caecal microbiota diversity and richness were not affected by broiler line, or its interaction with dose.

It has long been thought that selection for growth rate negatively affects aspects of bone mineralisation (Williams et al., 2004, 2000). In the present study, tibias and femurs of S line birds yielded more ash weight proportionally to their BW across the two slaughter points apart from femur ash weight which was initially similar between the two lines. These changes likely reflect the influence of the selection criteria of the maternal lines of the F line on body conformation traits. It has been proposed that improved mineralisation is achieved at lower growth rates due to the increased capacity of the skeleton to adapt to the increasing body mass (Brickett et al., 2007; Williams et al., 2004). Comparisons between selected fast-growing broilers with unselected ones demonstrated that several parameters are superior to the former in comparison to the latter when data were expressed in absolute values (McDevitt et al., 2006). However, as bones are components of body weight at increasing body weights concomitant increases in some minerals deposited and on bone breaking strength are expected to increase. More recently, comparison of fast and slow growing subpopulations from a randomised population indicated that almost all bone mineralisation measurements were greater in the slow-growing population when expressed per unit of BW at dissection at six weeks of age (Shim et al., 2012). Furthermore, phenotypic correlations within the same subpopulation indicate that growth rate is negatively associated with BBS and ash percentage (González-Cerón et al., 2015). Herein, percentage tibia and femur ash were similar among the two genotypes indicating similar rates of hydroxyapatite formation over the grower phase, which is indicative of bone maturation. On the other hand, femur BBS was smaller for birds of the F line at d26 of age which bears implications on their ability to tolerate the stresses applied to their long bones under the influence of high growth rates.

There are several reasons why differences between lines were absent under the hypothesis tested in relation to resistance and tolerance. There is a consensus that single trait selection in earlier genetic schemes focused on high productivity, such as increased weight gain and egg production, may have lead to unwanted consequences for traits that were not selected for, leading to the creation of less robust phenotypes with altered immune functions and decreased resistance and tolerance to infections (Havenstein et al., 2003; Hocking, 2014; van der Most et al., 2011). This would explain, to a large extent, previously described negative correlations between selection for performance and disease susceptibility (Rauw et al., 1998). In addition, experimental genetic selection in poultry for disease resistance has been based on immunity to a specific infectious agent, such as Marek’s disease or avian leukosis virus, or to a vaccine (Zekarias et al., 2002). This may have resulted in the formulation of inbred populations resistant to specific pathogens exhibiting lower resistance to others (Swaggerty et al., 2009). Attempt to draw comparisons in inbred lines, concerning the effect of growth rate, is therefore problematic. In addition, natural variation in antibody response, or different immune status of the heritage lines, could be driving the immune response observed when comparing different commercial broiler lines rather than selection for production traits *per se*. In the present study, the two genotypes originated from the same paternal lines, bred under identical husbandry conditions to account for these factors of variation. Our focus was not to look at immune pathways, but at the impact of a given pathogen on resistance and tolerance, and by proxy to account for their immunocompetence. It has been previously shown that selection for cellular and humoral immune responsiveness is possible without compromising growth, which may be related to the fact that relative energetic and protein requirements of immune responses are smaller than the ones of growth (van der Most et al., 2011). In the modern poultry industry, multi-trait selection is followed, encompassing functional traits in the selection programmes (Hocking, 2014) which allows for improvements in performance and health-related traits as part of a balanced breeding program (Kapell et al., 2012). On the other hand, the absence of a trade-off between growth rate and resistance may be related to the nature of the immune response evoked in the present host-pathogen model. The acute stage of a primary infection with *E.maxima* is predominated by Th-1 type immune responses (Cornelissen et al., 2009; Hong et al., 2006) which may be more preserved under different growth rates than Th-2 type immune responses evoked by nematode infections, where resistance has been shown to be sensitive to selection for growth rate (Coltherd et al., 2009; Zaralis et al., 2008). In the former case the immune response may be prioritized over growth due to the more severe pathology induced by intracellular pathogens and the necessity to effectively eliminate the infection to ensure host survival (Klasing, 2004). However, currently there is no experimental evidence to support this conjecture.

To summarise, faster growth rates did not lead to reduced resistance or tolerance to infection with *E. maxima*. Ranger Classic is a relatively new genotype destined for slow-growing broiler markets (Aviagen personal communication). The nutritional specifications for this line are not different from Ross 308, albeit they are expected to be lower due to the lower growth rate. Pathogen-induced anorexia may be sensitive to dietary nutrient adequacy, and this has implications for the differential response of fast *vs* slow growing genotypes (Kyriazakis, 2010). In addition, future studies should look into different coccidial species, such as *E. acervulina* and *E. tenella,* which evoke differential immune responses to that of *E. maxima* (Cornelissen et al., 2009).
